# Ectopic Reconstitution of a Spine-Apparatus Like Structure Provides Insight into Mechanisms Underlying Its Formation

**DOI:** 10.1016/j.cub.2024.11.010

**Published:** 2024-12-02

**Authors:** Hanieh Falahati, Yumei Wu, Mumu Fang, Pietro De Camilli

**Affiliations:** 1 Departments of Neuroscience and of Cell Biology, Howard Hughes Medical Institute, Program in Cellular Neuroscience, Neurodegeneration, and Repair, Yale School of Medicine, New Haven, CT 06510, USA.

## Abstract

The endoplasmic reticulum (ER) is a continuous cellular endomembrane network that displays focal specializations. Most notable examples of such specializations include the spine apparatus of neuronal dendrites, and the cisternal organelle of axonal initial segments. Both organelles exhibit stacks of smooth ER sheets with a narrow lumen and interconnected by a dense protein matrix. The actin-binding protein synaptopodin is required for their formation, but the underlying mechanisms remain unknown. Here, we report that the spine apparatus and synaptopodin are conserved from flies to mammals, and that a highly conserved region of this protein is necessary, but not sufficient, for its association with ER. We reveal a dual role of synaptopodin in generating actin bundles and in linking them to the ER. Expression in non-neuronal cells of a synaptopodin construct constitutively anchored to the ER is sufficient to generate stacked ER cisterns resembling the spine apparatus. Cisterns within these stacks are molecularly distinct from the surrounding ER and are connected to each other by an actin-based matrix that contains proteins also found at the spine apparatus of neuronal spines. Our findings shed light on mechanisms governing the biogenesis of this peculiar structure, and represent a step toward understanding the elusive properties of this organelle.

## eTOC Blurb

The spine apparatus is an enigmatic specialization of the smooth ER in neuronal dendritic spines. Falahati et al. engineer synthetic spine apparatus-like structures (SALs) in non-neuronal cells, showing the role of synaptopodin-induced F-actin networks in shaping this organelle. The SALs are continuous with the ER but molecularly distinct.

## Graphical Abstract

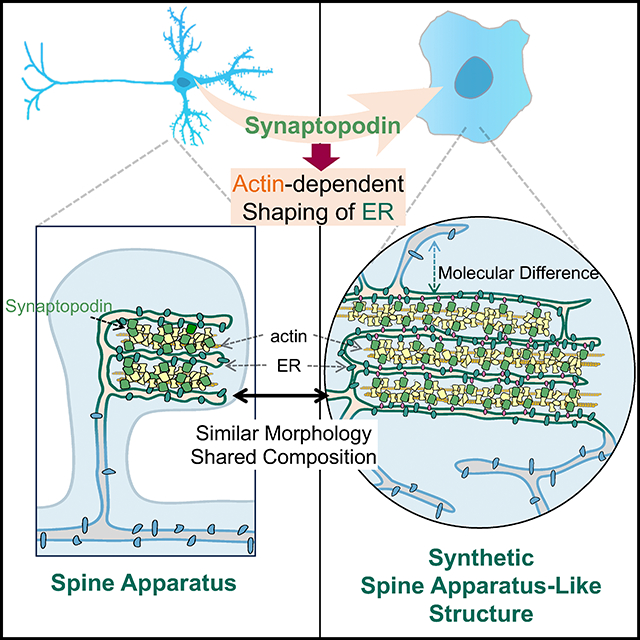

## INTRODUCTION

The endoplasmic reticulum (ER) is represented by an interconnected endomembrane system which extends into all regions of the cell cytoplasm^1–3^. However, it is organized in morphological and functional distinct domains, more so in specialized cells such as neurons^3–10^. A most notable example of specialized ER subdomain is the spine apparatus, strategically positioned in dendritic spines near synapses^11,12^. This structure is represented by a stack of interconnected flat smooth ER cisterns with a narrow lumen, which are separated from each other by a dense proteinaceous matrix and are connected to the bulk of the ER via a narrow tubule that travel along the spine’s stalk^3,12–14^. A morphologically similar ER specialization, called the cisternal organelle, can be observed at axonal initial segments^15,16^. Little is known about the functions of these ER subdomains, although there is evidence that the spine apparatus may have a role in synaptic plasticity^17–22^. A function in Ca^2+^ storage and Ca^2+^ signaling has been proposed^21,23,24^, although the contribution of its peculiar shape to such function remains unclear. Alterations in the morphology of the spine apparatus have been reported in response to long-term potentiation^25^ and pathological conditions^26–29^. While a number of studies have investigated the morphogenesis of ER subdomains like rough ER sheets and smooth tubules^30–33^, mechanisms underlying the formation of the spine apparatus and the cisternal organelle remain elusive.

Previous reports have shown that synaptopodin, a protein selectively concentrated in the spine apparatus and cisternal organelle in the brain, is required for the formation of these structures^16,17^. Synaptopodin is an actin-binding protein and, accordingly, the spine apparatus and cisternal organelle are localized in actin rich regions of dendritic spines and axonal initial segments^14,34–37^. Moreover, to discover other proteins associated with these structures, we had carried out a search of protein neighbors of synaptopodin using *in vivo* proximity proteomics and this approach identified several actin-related proteins^14^. These findings suggested a major role of actin and an actin-related machinery in the generation of the spine apparatus.

Formation of the spine apparatus and cisternal organelle involves several steps. Synaptopodin, which lacks a transmembrane region, must associate directly or indirectly with the ER. The ER, which typically consists of tubules in dendrites, must expand into sheets. These ER sheets need to be stacked on top of each other via a dense intervening matrix and this process must correlate with a narrowing of their lumen. The contributions of synaptopodin and synaptopodin neighbors to these steps, remain open questions in neuronal cell biology.

Goal of this study was to begin addressing these questions. Our findings reveal the evolutionary conservation of synaptopodin and of the spine apparatus from *Drosophila melanogaster* to mammals. We identify a highly conserved region within synaptopodin that is necessary for its association with ER. Remarkably, expression in COS-7 cells of a synaptopodin fusion protein directly anchored to the ER is sufficient to induce the formation of spine apparatus-like structures. Our findings suggest that interactions between synaptopodin and an actin-rich cytomatrix drive the formation of these organelles. Ectopic spine apparatus-like structures generated in non-neuronal cells represent a powerful model system to gain further insight into mechanisms that generate these specialized and poorly understood ER-based organelles.

## RESULTS

### Synaptopodin can crosslink ER to actin filaments in neurons.

**Figure 1.**
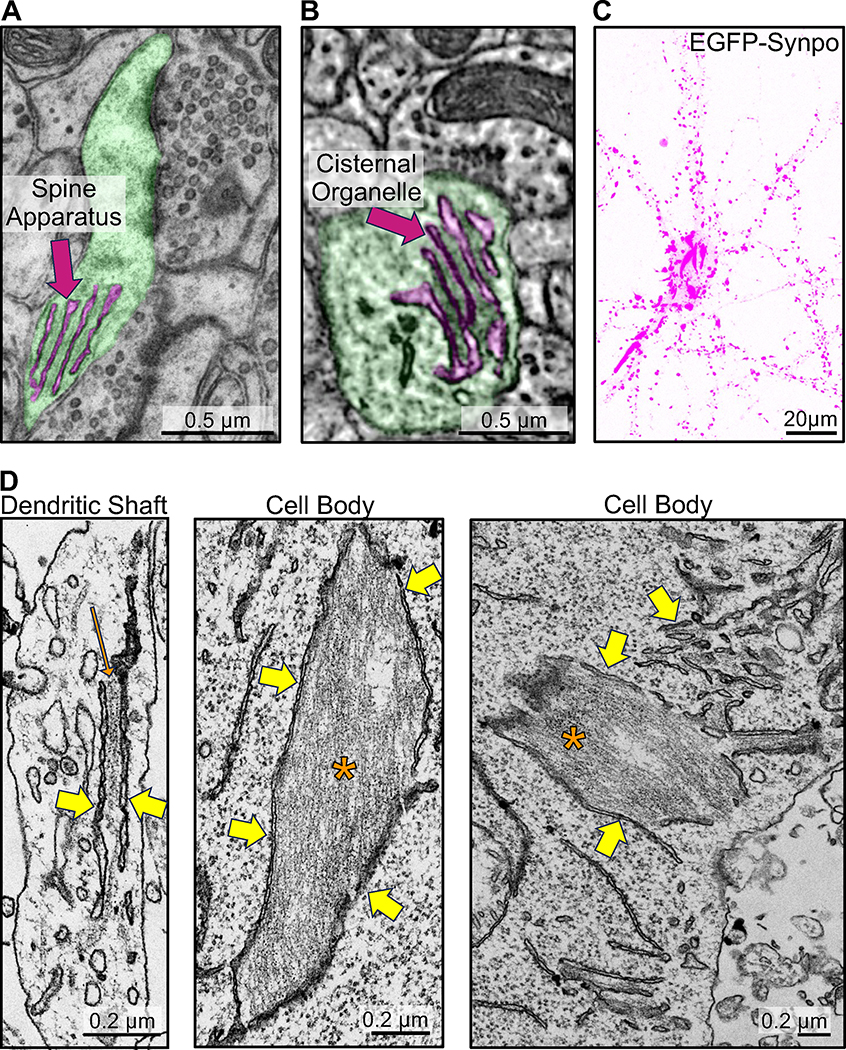
Synaptopodin mediated recruitment of actin filaments to the ER in neurons. **A.** TEM image of a pseudocolored dendritic spine (green) with spine apparatus (magenta). **B.** SBF-SEM image of a pseudocolored cisternal organelle (magenta) at an axonal initial segment (green) (image from microns-explorer.org). **C** and **D.** Fluorescent image (**C**), and TEM (**D**) of a cultured mouse hippocampal neuron infected with AAV2/9 encoding EGFP-synaptopodin. Note in C large accumulations of synaptopodin in the central region of the cells in addition to the punctate fluorescence in dendrites. Many of smaller puncta along dendrites correspond to the expected accumulations of synaptopodin in the spine apparatus of dendritic spines. Yellow arrows in **D** point to the ER and one orange arrow and asterisks point to actin bundles. See also Figure S1 and Figure S2.

The two neuron-specific ER specializations positive for synaptopodin, the spine apparatus and the cisternal organelle, share special morphological features. They are composed of stacks of smooth ER sheets which have a very narrow lumen and are connected to each other by a proteinaceous dense matrix (Figure 1A–B)^11,12^. Concentrations of both endogenous and exogenous synaptopodin - as revealed by immunofluorescence and by expression of fluorescently tagged synaptopodin respectively - are also observed in dendritic spines and axonal initial segments in cultured mouse hippocampal neurons^14,35^ (Figures 1C, S1A–C). Fluorescent exogenous (and thus overexpressed) synaptopodin also accumulated in spots or elongated structures (up to several micron long) in dendritic shafts and perikarya (Figure 1C). The nature of these synaptopodin accumulations was analyzed by EM. To this aim, cultured DIV7 neurons were transduced with AAV2/9 viruses encoding EGFP-synaptopodin and cells showing high abundance of these fluorescent structures were selected for analysis by correlative light and electron microscopy (CLEM). EM observation revealed that the synaptopodin-positive accumulations were represented by bundles of filaments with the expected morphology of F-actin sandwiched between ER cisterns (Figures 1D, S2). In some cases, only a thin layer of actin separated ER cisterns, thus generating structures reminiscent of ER stacks of the spine apparatus. Somewhat similar synaptopodin-positive structures- pairs of ER cisterns connected by a synaptopodin-positive layer - have been observed to form next to neuronal cell bodies in response to high neuronal activity^38^. These results show that synaptopodin, when expressed in neurons, not only can bundle F-actin, as shown previously^14,34,36^, but can also crosslink F-actin bundles to the ER. They support a key role of synaptopodin in the formation of the spine apparatus and cisternal organelles, which are ER-based structures.

### Synaptopodin can crosslinks ER to F-actin bundles also in fibroblasts.

**Figure 2.**
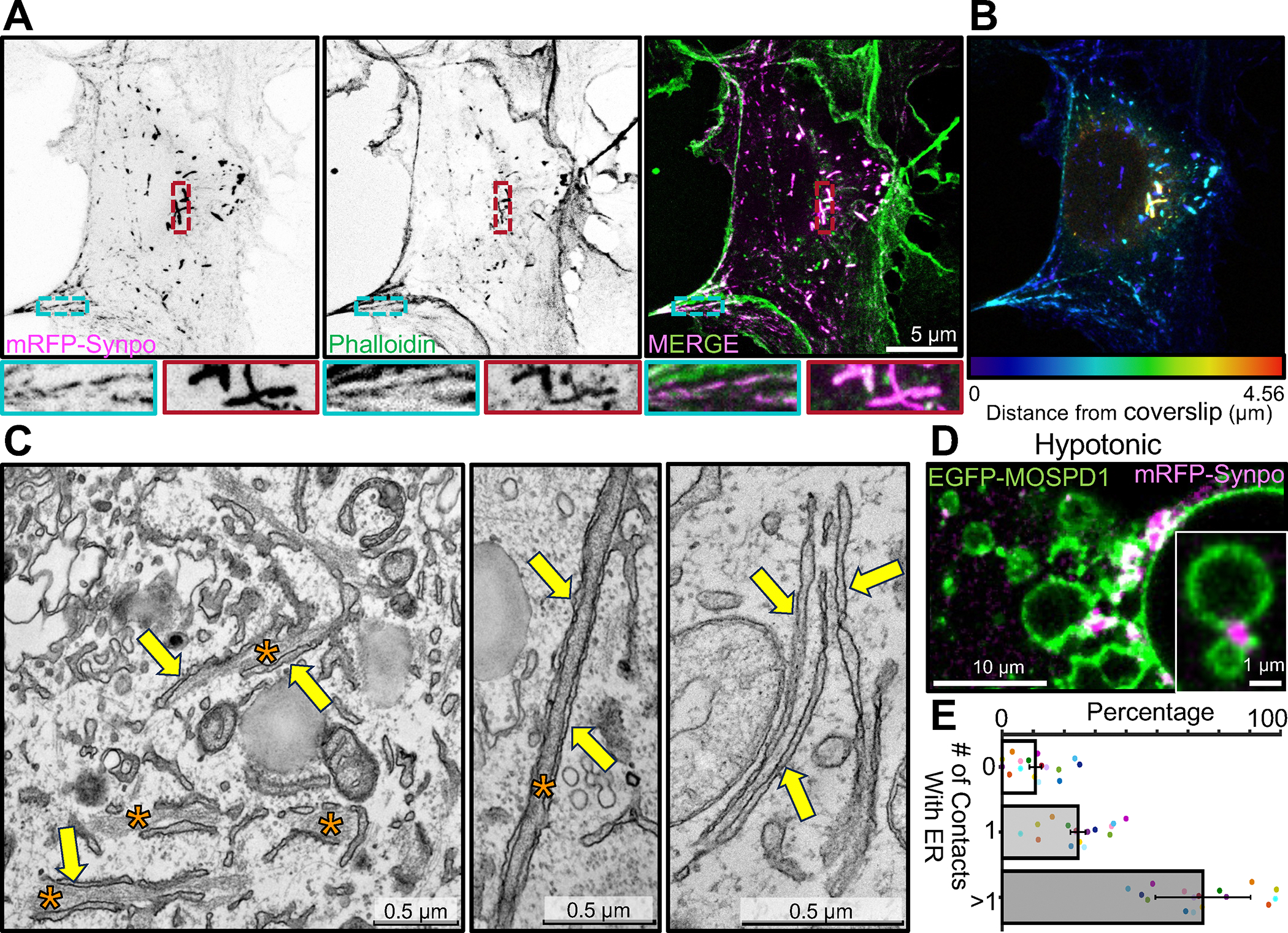
Localization of exogenous synaptopodin in COS-7 cells both on stress fibers and on actin-rich structures associated with ER. **A.** COS-7 cells expressing mRFP-synaptopodin displaying strong overlap of the mRFP fluorescence with phalloidin staining. High magnification views of a stress fiber (blue) and a non-stress fiber linear assembly (red) are shown at the bottom. **B.** mRFP-synaptopodin signal from A is shown with color-coding based on the distance from the coverslip. Stress fibers are closer to coverslip while non-stress fiber actin assemblies are present anywhere within the cell. **C.** TEM of a cell expressing mRFP-synaptopodin showing actin bundles (orange asterisks) sandwiched between ER sheets (yellow arrows). **D.** COS-7 cells expressing mRFP-synaptopodin (magenta) and the ER protein EGFP-MOSPD1 (green) were exposed to hypotonic conditions. The accumulation of synaptopodin at the interface between ER elements reveals its direct or indirect association with the ER membrane. **E.** Percentage of mRFP-synaptopodin puncta with or without association with ER membrane in COS-7 cells exposed to hypotonic conditions is shown. Data from different cells are shown as a dot with different color. See also Video S1.

We further investigated the relationship between synaptopodin, actin and the ER by expressing fluorescently tagged synaptopodin (mRFP- or EGFP-synaptopodin) in COS-7 cells, a fibroblastic cell line whose flat morphology facilitates the analysis of these relationships by fluorescence microscopy. As reported^14,34^, strong colocalization of mRFP-synaptopodin with F-actin (phalloidin staining) was observed in these cells (Figure 2A). A subset of these synaptopodin- and actin-positive elements had the appearance of stress fibers. Another subset of them were thick linear inclusions generally located deep in the cytoplasm and away from the plasma membrane (Figure 2A; see color coding of depth in 2B). At EM observation these structures were found to correspond to bundles of actin sandwiched between tightly apposed ER cisterns (Figure 2C) similar to those observed in neurons overexpressing synaptopodin (Figure 1D). No such structures were observed in non-transfected COS-7 cells.

The tight attachment of these synaptopodin-positive elements to the ER was further supported by acute exposure of cells to drastic hypotonic conditions, an approach previously used to examine ER contacts with other structures^39,40^. Upon hypotonic shock, the ER rapidly vesiculates, but many of its contacts with other structures persist for some time and can be visualized as the cell swells and undergoes lysis. For these experiments cells were transfected not only with mRFP-synaptopodin, but also with EGFP-MOSPD1 as a marker of the ER^41^ (Figure 2D, Video S1). After 60 minutes from the beginning of the hypotonic shock, 90% (± 2%) of the synaptopodin positive elements were still in contact with the ER, confirming the occurrence of a link between them (Figure 2E). Moreover, 65% (± 15%) of them were at contacts between ER vesicles consistent with their role in crosslinking ER cisterns before the hypotonic lysis.

We conclude that even in fibroblasts synaptopodin not only can bundle actin, but can also link such bundles to the ER, implying the presence in this protein of sites that directly or indirectly bind the ER membrane.

### Synaptopodin has multiple actin binding sites.

**Figure 3.**
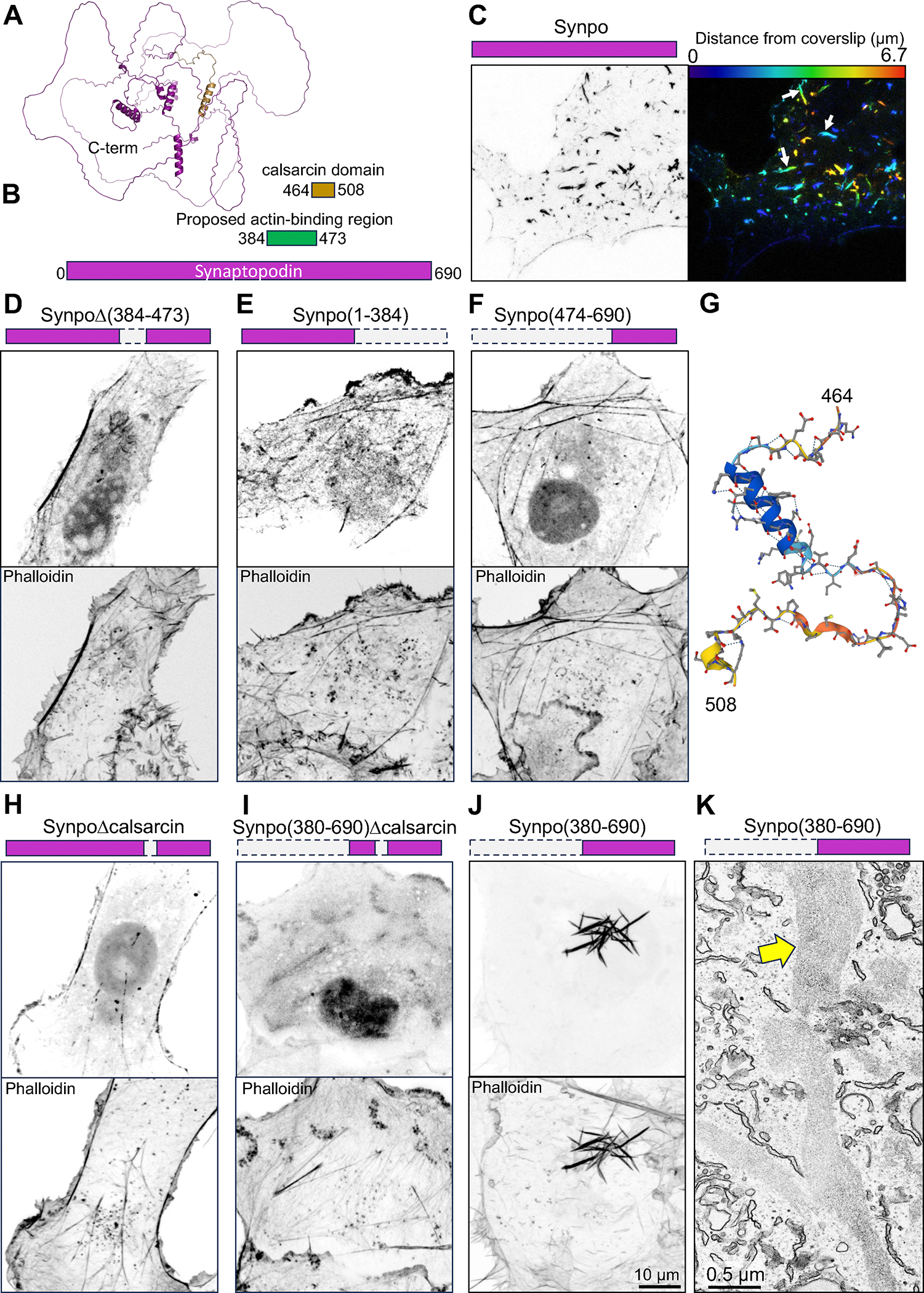
Synaptopodin regions involved with F-actin and ER binding. **A.** Predicted structure of mouse synaptopodin (accession number: Q8CC35–3) based on Alphafold3. The calsarcin domain is shown in gold. **B.** Domain representation of synaptopodin, showing in green the previously reported actin binding region^42^ and its partial overlap with the calsarcin domain (gold). **C.** COS-7 cell expressing fluorescently tagged full-length synaptopodin and showing the prominent accumulation of synaptopodin inclusions (arrows) that represent actin-ER assemblies. The synaptopodin signal is shown in gray scale (left), and color-coded based on distance from coverslip (right). **D – F.** COS-7 cells expressing the indicated deletion constructs of synaptopodin (magenta color) lacking the reported actin binding region showing that all of them still partially colocalize with F-actin as indicated by phalloidin staining. **G.** Predicted structure of the calsarcin region of synaptopodin based on Alphafold. **H -J.** COS-7 cells expressing synaptopodin deletion constructs showing that the construct including the calsarcin domain (**J**), but not the ones excluding this domain (**H** and **I**) induce the formation of synaptopodin inclusions (see Figure 2). **K.** EM image of a COS-7 cell expressing Synpo(380–690) showing that unlike wildtype synaptopodin, this construct bundles actin but does not induce their association with the ER (compare with Figs. 1D, 2C and S2). A yellow arrow points to a Synpo(380–690)-induced inclusion (massive actin bundle). See also Figure S3.

Synaptopodin is not predicted to have major folded domains (Figure 3A). In order to identify the region(s) within synaptopodin responsible for its property to crosslink actin bundles to the ER, we generated several truncation mutants of this protein and tested them for their property to generate the actin-rich ER-associated structures described above. Previous work showed that residues 384–473 include the actin binding region of synaptopodin^42^ (Figure 3B). We found that deletion of residues 384–473 did not eliminate its property to associate with F-actin, suggesting the presence of additional actin binding sites (Figure 3C–D). However, this mutant no longer formed the F-actin positive inclusion that reflect the ER-associated actin bundles (see Figure 2A). Both regions upstream (residues 1–384) or downstream (residues 474–690) retained their ability to localize to stress fibers (Figure 3E–F), confirming that synaptopodin can directly or indirectly interact with F-actin through multiple regions.

We observed that the fragment of synaptopodin previously identified as the actin binding region^42^ and found by us to be required for the formation of ER-associated inclusions, i.e. the fragment comprising a.a. 384–473, partially overlapped with a 45 a.a. stretch classified as calsarcin domain in Pfam (a.a. 464–508) (Figure 3B, G). Deletion of these residues from full length synaptopodin or its C-terminal fragment (a.a. 380–690) abolished the F-actin positive cytoplasmic inclusions (Figure 3H–I), while such inclusions were prominent in cells expressing the C-terminal fragment comprising this region (Figure 3J). However, the cytoplasmic inclusions generated by the C-terminal fragment were different from those generated by full length synaptopodin in that they did not associate with ER, as shown by EM (Fig 3K). In addition, a construct comprising the calsarcin domain fused to the N-terminal of EGFP was cytosolic, indicating that this domain is not sufficient to anchor a protein to the ER. These results indicate that the calsarcin domain is necessary for the formation of ER-associated cytoplasmic inclusions, but it is not sufficient for the association of synaptopodin with ER. The precise nature of the interaction of synaptopodin with the ER remains to be elucidated.

The region of synaptopodin defined in databases as calsarcin domain comprises an alpha-helix followed by a hairpin (Figure 3G). The alpha-helix share similarity to an alpha-helix of myozenin^43^ (also known as calsarcin, hence the name of the domain) (Figure S3A). Interestingly, myozenin, like synaptopodin, binds Pdlim family proteins^44^, which include Pdlim7, a synaptopodin interactor^14^, although myozenin does not use the calsarcin domain for such binding^44^. The calsarcin domain is also highly conserved in the synaptopodin paralogues synaptopodin 2 and synaptopodin 2-like^45^, and extends to the hairpin region (67% similarity, Figure S3A). Moreover, when expressed in COS-7 cells, fluorescently tagged synaptopodin 2-like protein also localizes on internal F-actin inclusions similar to those generated by synaptopodin (Figure S3B). Based on public gene expression databases (e.g. https://tabula-sapiens-portal.ds.czbiohub.org), synaptopodin 2 and synaptopodin 2-like have a similar expression pattern as synaptopodin in human brain, but are mainly absent from mouse neurons. This likely explains why in mice the lack of synaptopodin is sufficient to abolish presence of spine apparatus and cisternal organelles.

### Synaptopodin and the spine apparatus are present in flies.

**Figure 4.**
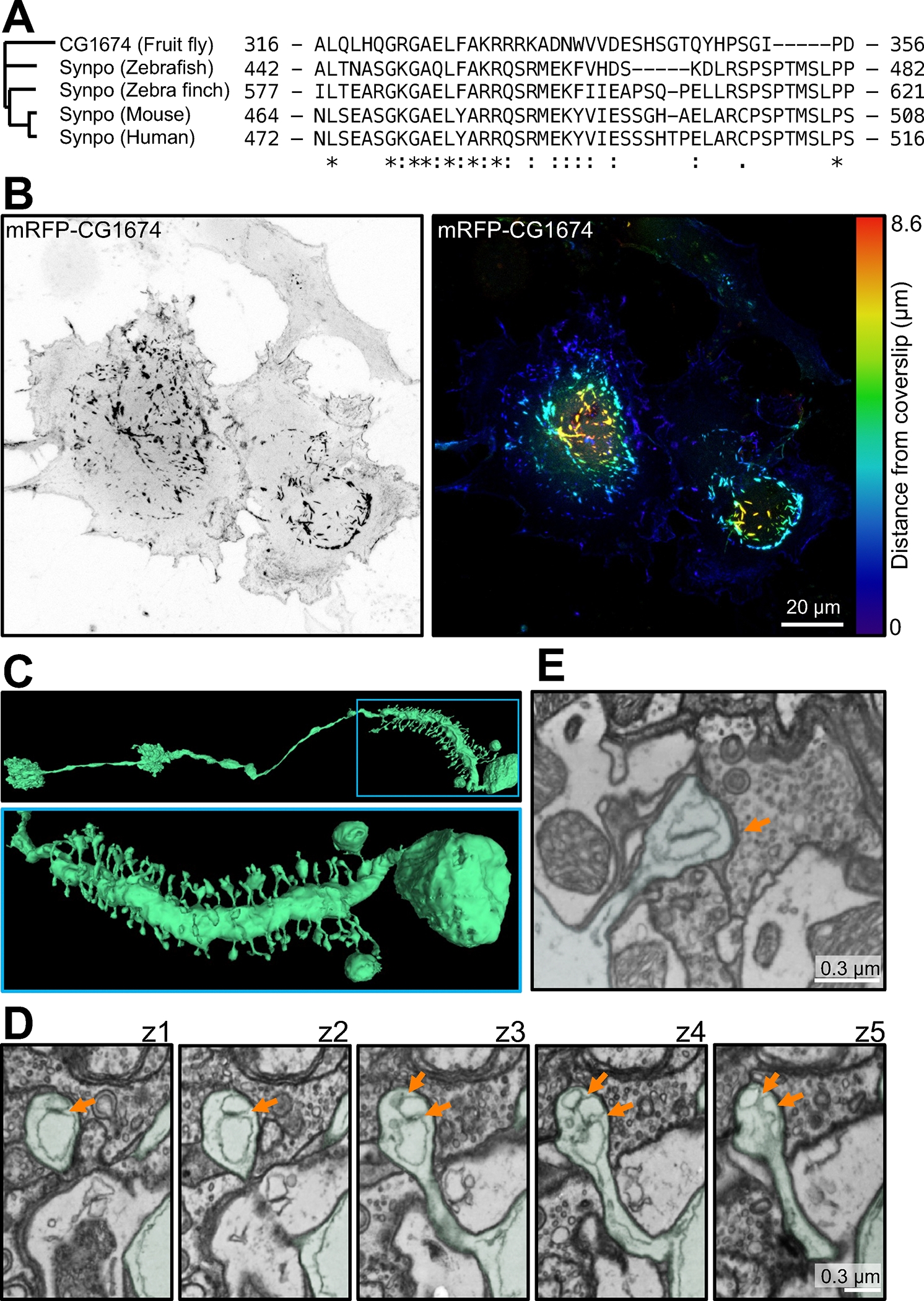
Presence of synaptopodin and spine apparatus in *Drosophila melanogaster*. **A.** Residues 464–508 of mouse synaptopodin (calsarcin domain) are highly conserved among synaptopodin orthologues in vertebrates and *D. melanogaster* orthologue, CG1674. **B.** COS-7 cell expressing the fluorescently tagged *D. melanogaster* synaptopodin orthologue. A prominent accumulation of CG1674 inclusions, similar to those observed in cells expressing fluorescently tagged mouse synaptopodin (See Fig 2 and 3C), is visible. The CG1674 signal is shown in gray scale (left), and color-coded based on distance from coverslip (right). **C.** *D. melanogaster’s* L1 neuron reconstructed from 3D second generation TEM camera array (TEMCA2) images (from FlyWire^50^) showing spines along its major process. Low and high magnification views are shown at the top and bottom, respectively, **D.** TEM sequential optical sections from a spine of the neurons shown in **C**, revealing ER elements closely apposed via an intervening density (orange arrows) in the spine head. This structure is reminiscent of a spine apparatus with dilated ER cisterns, possibly due to preparation artifacts. **E.** Another example of a dendritic spine of *D. melanogaster* containing a structure reminiscent of a spine apparatus, but with a dilated ER. See also Figure S3.

To delve deeper into the contribution of synaptopodin to spine apparatus formation, we explored its occurrence throughout evolution. Analysis of the evolutionary tree of synaptopodin using Panther^46^ revealed that one of its early orthologues is CG1674 in *Drosophila melanogaster*. CG1674 is an actin binding protein^47^, and its calsarcin domain shares 47% similarity with that of human synaptopodin (Figure 4A). Accordingly, expression of mRFP-CG1674 in COS-7 cells resulted in the formation of F-actin inclusions localized deep in the cytoplasm similar to those observed upon expression of mammalian synaptopodin (Figure 4B–C). Moreover, coexpression of mRFP-CG1674 with mouse synaptopodin (EGFP-synaptopodin) resulted in their precise colocalization (Figure S3C). Since the presence of spine apparatus was only reported in mammals^11,12,48,49^, we searched the entire brain electron microscopy dataset of *D. melanogaster*, FlyWire (https://flywire.ai^50^). Interestingly, while most *D. melanogaster* neurons lack spines, L1 neurons have protrusions that share morphological similarities with dendritic spines of vertebrates (Figure 4C) and in such spine-like protrusions, the presence of two to three ER sheet elements closely apposed to each other by an intervening proteinaceous density can be observed (Figure 4D–E). While such ER elements have a wide lumen, in contrast to ER elements of the mammalian spine apparatus, it remains possible, that such width may reflect a dilation due to fixation conditions.

Together, these observations imply that synaptopodin and the spine apparatus may be conserved from flies to mammals.

### A Spine-Apparatus-Like structure (SAL) is reconstituted in COS-7 cells.

The results and observations reported above reveal that synaptopodin can bind and bundle actin and also link actin to the ER. These properties suggest that synaptopodin could mediate the close apposition of ER elements observed in the spine apparatus via an intervening actin matrix. However, a major difference observed between the spine apparatus and the structures induced by synaptopodin overexpression in both neurons and COS-7 cells is the thickness of the actin bundles separating the ER cisterns. Such greater thickness is most likely due to actin crosslinking by synaptopodin not in contact with the ER. What limits actin bundling in dendritic spines remains unclear.

**Figure 5.**
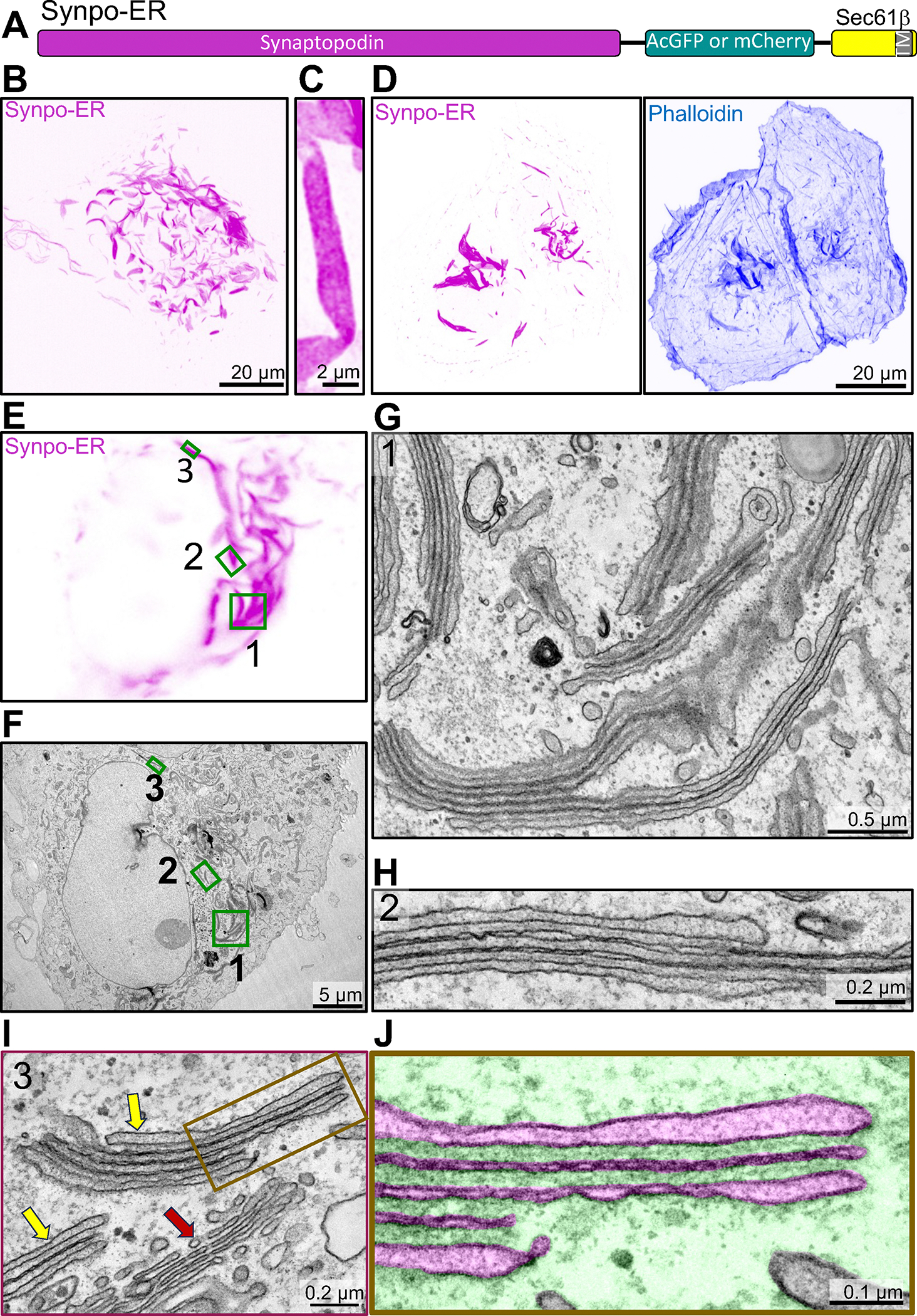
Generation of spine apparatus-like structures (SALs) in COS-7 cells. **A.** Diagram of the synaptopodin construct anchored to the ER (Synpo-ER) by its fusion to Sec61β, which is embedded in the ER by a C-terminal transmembrane region. **B.** Confocal image of a COS-7 cell expressing Synpo-ER. **C.** High magnification view of a SAL as imaged by an AiryScan confocal microscope. **D.** Presence of F-actin in SALs as indicated by phalloidin staining. **E** and **F.** Light microscope image (**E**) of a COS-7 cell expressing synaptopodin with its corresponding EM image (**F**). High magnification EM images of the regions 1 – 3 framed by green rectangles in fields D and E are shown in **G** - **I**, respectively. Moreover, a portion of the SAL in **I** is shown at higher magnification in **J** with the ER lumen pseudocolored in magenta and the cytosolic space in green. SALs are represented by ER stacks with morphological features similar to those of the spine apparatus and of the cisternal organelle. Red and yellow arrows in I point to the Golgi complex and to two SALs, respectively. See also Figure S4 and Video S2.

We explored what would happen if we restricted synaptopodin binding and bundling of F-actin exclusively to when it is in contact with the ER, by anchoring it to the ER via a transmembrane region. This was achieved by using a construct (synaptopodin-ER, Figure 5A) in which synaptopodin (as a fluorescent fusion protein) is fused to the N-terminus of Sec61β, an ER resident protein anchored to this organelle through a C-terminal transmembrane region^51^. As shown previously^14^, expression of this chimera in COS-7 cells resulted in the formation of elongated inclusions (Figure 5B–D) that were positive for actin - as shown by phalloidin staining (Figure 5D)- and bore a resemblance to those induced in the cell bodies of neurons (Figure 1C) and COS-7 cells (Figure 2A) by synaptopodin overexpression. In cells with high levels of synaptopodin-ER such structures were extremely abundant (Figures 5B and D).

Strikingly, CLEM of synaptopodin-ER expressing cells (Figure 5E–J) showed that the fluorescent signal of synaptopodin-ER corresponded to ER sheets stacked together with only a thin dense matrix separating them (Figures 5F–J, S4A–B). These stacks, as observed by correlative light and FIB-SEM, often extended over several micrometers in length (Figure S4C and Video S2). FIB-SEM also showed that cisterns were continuous with the tubular ER (Video S2). Within these stacks, ER sheets had a very narrow, nearly absent lumen, with the exception of the first and last cisterns. Accordingly, DsRed-KDEL was nearly excluded from these stacks in live cells (Figure S5), implying that most luminal ER proteins are likely excluded from these structures. Overall, the appearance of these ER stacks, which we will refer to henceforth as SALs (Spine Apparatus-Like) had key distinctive morphological features of the spine apparatus and cisternal organelle. Moreover, as in the spine apparatus, a dense matrix was observed between ER sheets (Figure 1A–B), although the spacing between the sheets (28.2 ± 0.8 nm) (Figure S4D) was narrower than in the spine apparatus (51 ± 2 nm) (Figure S4D). Correlative light and FIB-SEM showed that higher synaptopodin-ER fluorescent signal correlated with SALs that have higher number of ER sheets (Figure S4E, R = 0.6).

**Figure 6.**
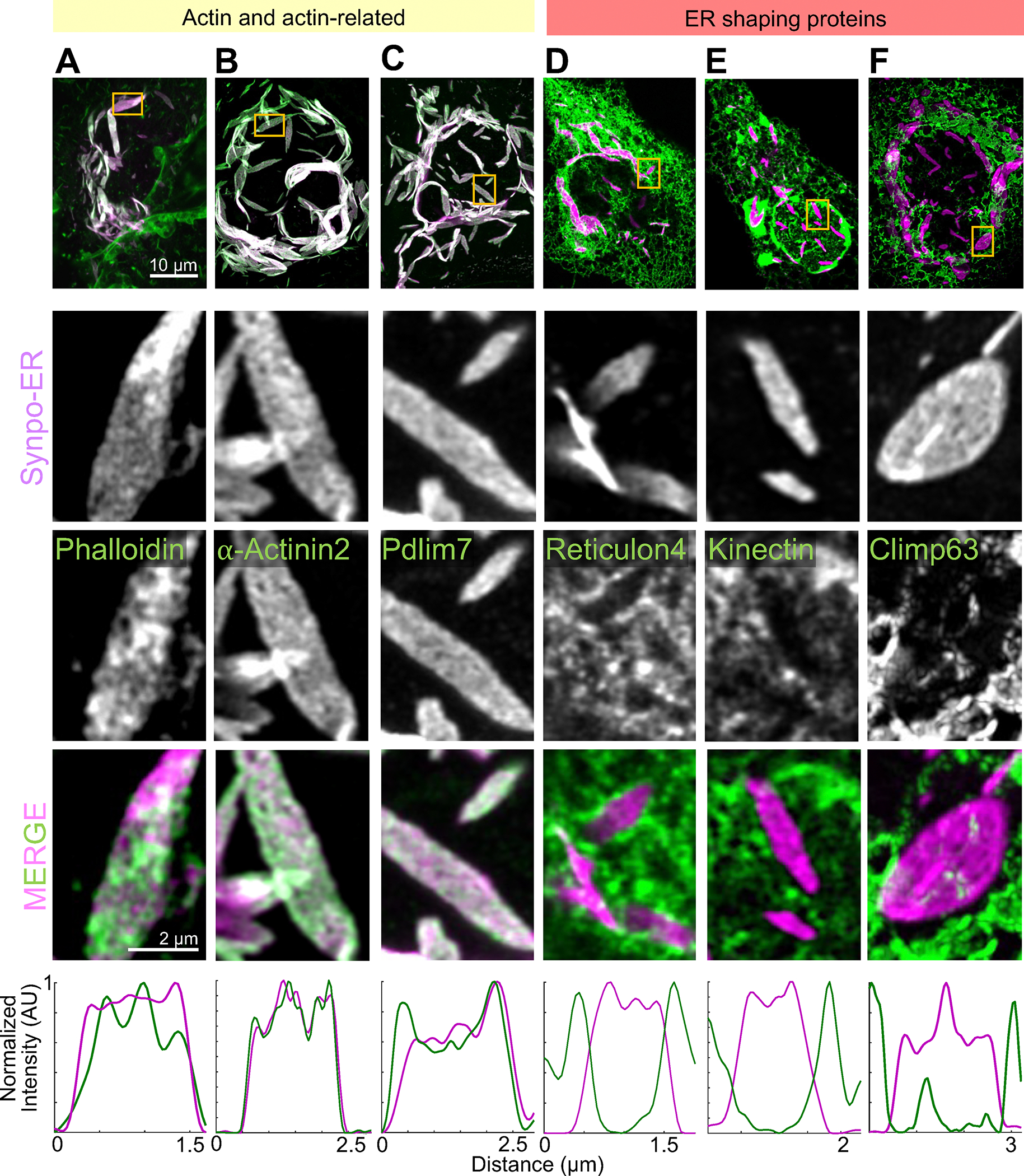
Presence of actin and actin-related proteins in SALs. **A-F.** Low (first row) and high (rows 2–4) magnification AiryScan images of individual SALs showing the localization of Synpo-ER and of the fluorescently tagged proteins indicated. Line scan plots are shown in the bottom. Reticulon 4-GFP is enriched at the edges of SALs and excluded from their flat portions. GFP-Kinectin and GFP-Climp63 are excluded from SALs. See also Figure S5.

Interestingly, housekeeping ER membrane proteins such as VAP-B, STIM1 and MOSPD1 are also excluded from these structures (Figure S5) suggesting that while SALs are continuous with the rest of ER, they are molecularly distinct subdomains. Another protein tested, GFP-Sec61β, however, was localized in SALs, indicating that not every ER protein is excluded from them (Figure S5). In addition to actin (Figure 6A), the proteins that we previously identified to localize to the spine apparatus in the brain, α-actinin 2, Pdlim7, Magi1, and Magi2, all localize to SALs^14^ (Figures 6B–C, S6), confirming that SALs share molecular components with the spine apparatus. We conclude that expression of synaptopodin-ER generates ER specializations that can provide insight into mechanisms underlying formation of the spine apparatus.

### Formation of SALs is driven by a crosslinked actin meshwork on the ER surface.

As SALs are represented by ER sheets, we examined the contribution to their structure of ER-shaping proteins. Reticulon 4-GFP and EGFP-Atlastin 1, which are known to localize to, and stabilize, positive curvatures in ER membranes^52–54^, localized to the edges of SALs and were excluded from their flat surfaces in live COS-7 cells (Figures 6D, S6). Lunapark, another ER shaping protein that localizes to membranes with negative curvature^55^ was enriched at hot spots which corresponded to discontinuities of the synaptopodin-ER signal within the sheets (Figure S6). These discontinuities likely correspond to the fenestrations^56,57^ exhibiting negative curvatures within the sheets as revealed by EM images of SALs (Figure S4A blue arrow). In contrast, the two sheet-forming proteins that we investigated, namely GFP-Kinectin (Figure 6E) and GFP-Climp63^54,58^ (Figure 6F), did not localize to the flat portion of SALs sheets, indicating that they do not play a role in their formation.

*En-face* views of ER cisterns of SALs stained with phalloidin revealed a dense meshwork of actin between them, supporting a model in which synaptopodin crosslinks cisterns via its multivalent binding to actin (Figure 6A). Moreover, as discussed above, actin binding proteins that we had previously found to be components of the spine apparatus, such as Pdlim7 and α-actinin 2^14^, colocalized with synaptopodin-ER when co-expressed with this protein in live COS-7 cells, suggesting that they are part of the dense matrix that connect cisterns of SALs (Figure 6B–C). In contrast, EGFP-MyosinIIA (Figure S6A) did not accumulate at SALs, indicating that actin connecting the ER sheets is not part of an actomyosin contractile network.

The ability of synaptopodin to form SALs if anchored to the ER was shared by the fly ortholog of synaptopodin, CG1674 (Figure S6B), consistent with its conserved role in the formation of the spine apparatus. Likewise, targeting to the ER the synaptopodin paralog, synaptopodin 2-like protein, and its longer isoform found in kidney podocytes, resulted in the formation of similar structures in COS-7 cells (Figure S6B). Since the spine apparatus is only present to neurons, some neuron-specific factor, yet to be identified, must be responsible for the property of synaptopodin to assemble cisternal stacks only in these cells. The property of proteins to generate SALs if anchored permanently to the ER is not a general feature of actin binding proteins. α-actinin 2, an interactor of both F-actin and synaptopodin^14,36,42^, did not show this property, as when α-actinin 2-GFP-Sec61β was expressed in COS-7 cells, its localization mirrored that of the general ER marker, DsRed-KDEL, a 27KD fluorescent protein which localizes to the ER lumen (Figure S6C–D).

**Figure 7.**
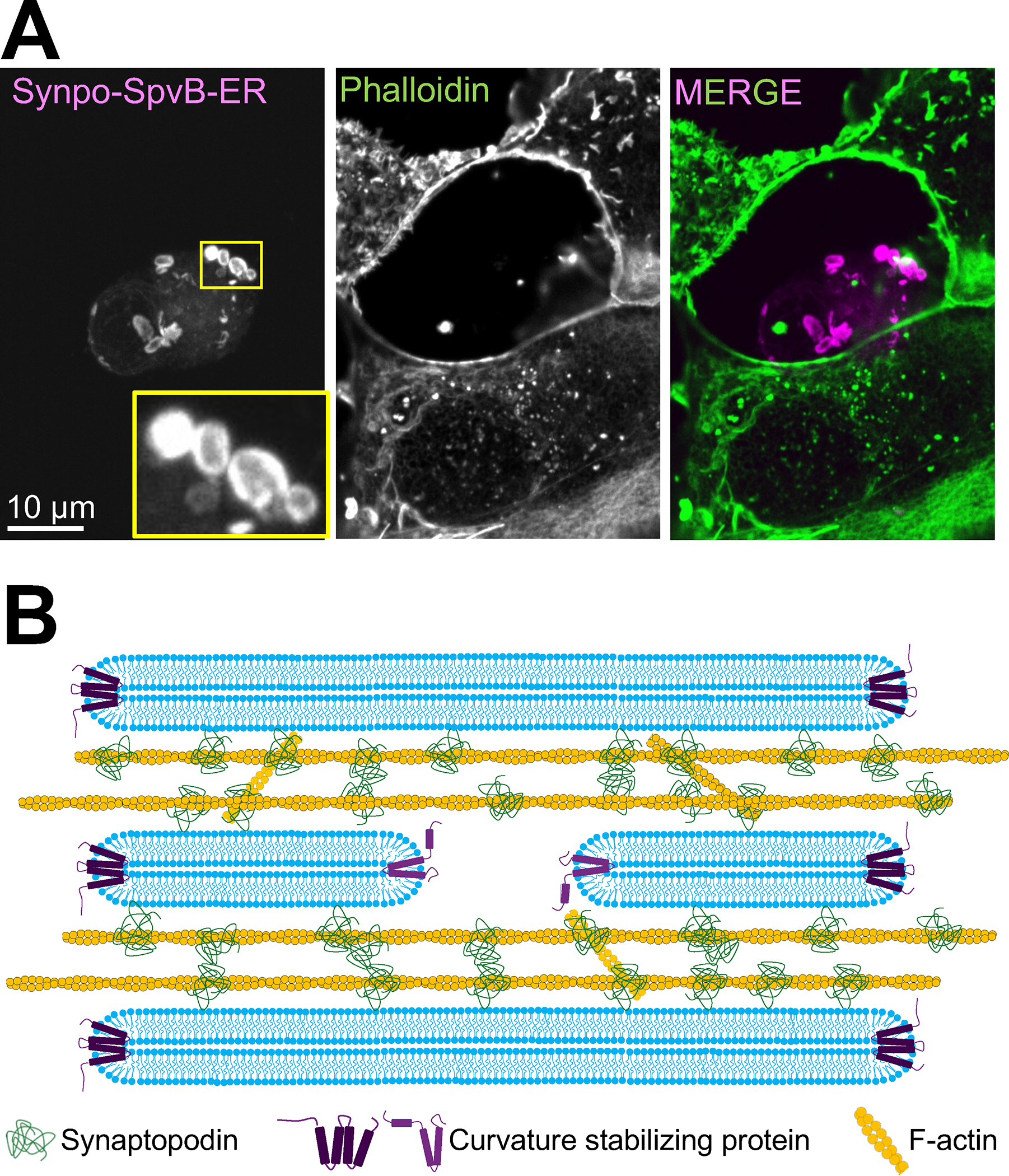
Proposed model for the formation of the spine apparatus and cisternal organelle. **A.** Expression of Synpo-SpvB-mCherry-Sec61β in a COS-7 cell results in the loss of all F-actin, including F-actin associated with ER-targeted synaptopodin, while the neighboring cells that do not express this fusion construct retain their normal actin cytoskeleton. The cell expressing Synpo-SpvB-mCherry-Sec61β lacks SALs and instead contains OSERs^61,62^, structures known to be induced by homotypic interaction between ER proteins. A zoomed-in portion of OSERs is shown in the inset. **B.** Synaptopodin associates with ER by an unidentified binding partner, and subsequently recruits and crosslinks an actin meshwork to this organelle. This process leads to the expansion and stacking of the ER sheets as well as to the narrowing of their lumen. See also Figure S6.

To further examine the role of actin in shaping SALs, we treated COS-7 cells expressing synaptopodin-ER with 5μM Cytochalasin D or 2μM Latrunculin A for 2 hours. After fixation and staining F-actin with phalloidin, we observed that while both drugs caused stress fibers to collapse, they failed to depolymerize F-actin associated with SALs (Fig S7E–F). Since both Cytochalasin D and Latrunculin A affect dynamic actin filaments, these results suggest that F-actin associated with SALs are stable within the 2hrs incubation time. To prevent F-actin assembly at SALs, we engineered a synaptopodin-ER fusion construct comprising the bacterial enzyme SpvB (Synaptopodin-SpvB-mCherry-Sec61b). This enzyme is known to act as a “genetically-encoded latrunculin”: it ribosylates monomeric actin and prevents its incorporation into filaments^59,60^. COS-7 cells expressing this construct did not stain with phalloidin and lacked SALs. Instead, in these cells the ER formed round OSER (organized smooth ER)-like structures which arise from weak homotypic interactions between ER proteins^61,62^ (Figure 7A). Based on these results, we propose that the expansion, stacking and distinct morphology of ER sheets at SALs is driven by the formation of an actin-based cytomatrix nucleated by, and bound to, synaptopodin at the interface of ER cisterns (Figure 7B).

## DISCUSSION

The spine apparatus and the cisternal organelle are two specialized subdomain of the ER that share morphological features and require the actin binding protein synaptopodin for their formation^16,17^. In this work we show that wild-type synaptopodin not only can crosslink and bundle actin, as previously reported^34,36,42^, but can also connect actin bundles to the ER in living cells, both in neurons and in non-neuronal cells. A highly conserved 45-amino-acid region within synaptopodin is necessary for its property to link actin to the ER, although this domain alone was not sufficient to target a protein to the ER.

We have further shown, using a heterologous system (COS-7 cells) that artificially anchoring synaptopodin to the ER by fusing it to Sec61β, which contains a C-terminal anchor^51^, results in structures (SALs) that resemble the spine apparatus in key morphological features and at least some molecular properties. Like the spine apparatus, SALs consist of stacks of cisterns with a very narrow lumen connected by a dense matrix that contains actin and actin binding proteins also found in the spine apparatus of neurons. The narrow lumen is not a fixation artifact, as live imaging of SALs in cells also expressing the luminal marker, DsRed-KDEL showed absence of this marker from SALs. Previously studied ER-sheet enriched proteins do not localize to SALs, indicating that known ER-sheet forming mechanisms do not apply to their formation and that cisterns apposition via an intervening non contractile actin-based matrix is sufficient to expand tubules into sheets and to narrow their lumen. Contractile and non-contractile actin meshworks are known to be implicated in the dynamics of subcellular organelles. Our finding reveals a new role of a non-contractile actin meshwork in membrane shaping.

The distinct molecular composition of SALs, despite their continuity with the entire ER network, suggests that the spine apparatus and cisternal organelle also differ in their protein content from the rest of ER. This distinct molecular composition might create a functionally specialized subdomain within ER, tailored to meet the specific needs of the dendritic spine and axonal initial segment. The enrichment in ER membranes of these structures with minimal luminal space suggests that membrane associated functions, including lipid signaling, may predominate. Such thinning of ER is also frequently observed in subsurface ER cisternae, suggesting that the functional distinction may potentially be expandable to other ER specializations.

## RESOURCE AVAILABILITY

### Lead contact

Requests for further information and resources should be directed to and will be fulfilled by the lead contact, Pietro De Camilli (pietro.decamilli@yale.edu).

### Materials availability

All recombinant plasmids generated for this study are available from the lead contact with a completed materials transfer agreement.

### Data and code availability

- All raw images reported in this paper will be shared by the lead contact upon request.
- This paper does not report original code.
- Any additional information required to reanalyze the data reported in this paper is available from the lead contact upon request.

## STAR METHODS

### EXPERIMENTAL MODEL AND STUDY PARTICIPANT DETAILS

#### Primary Neuronal Culture and Transfection

Hippocampi of P0-P1 C57BL/6J mice were dissected on ice in Hibernate-A media. Dissected hippocampi were then washed in ice cold Dissociation medium (final concentration: 5.8mM MgCl_2_, 0.252mM CaCl_2_, 10mM HEPES pH = 7.4, 1mM Pyruvic Acid, 81.7% Mg and Ca free HBSS) and immediately digested in Cysteine-activated Papain solution (17U/ml Papain, 20μg/ml DNase I, 2mg/ml of L-Cysteine Hydrochloride in Dissociation media) for 30 min at 37 °C. Samples were then washed in sequence with 10% FBS (in Dissociation media), Dissociation medium and Neurobasal-A medium supplemented with 2% B27 and 2mM L-Glutamax. Subsequently, 120–150K cells were plated on the glass bottom of MatTek plates coated with Poly-D-Lysine (1mg/ml) in 150μl of Neuronal Growth Medium [Neurobasal-A supplemented with 2% B27, 2mM L-Glutamax, 15% glial enriched medium (Neurobasal-A supplemented with B27 collected from a culture of DIV7+ mouse glial culture) and 10% cortical enriched medium (Neurobasal-A supplemented with B27 collected from DIV7+ mouse neuronal culture)]. 4–16hrs after plating, 2ml of Neuronal Growth Medium was added to each plate and an additional 0.5ml of Neuronal Growth Medium was added to each plate every 3–4 days afterwards. Hippocampal neurons were transfected on DIV11–13 using the CalPhos Mammalian Transfection kit (Takara) according to manufacturer’s instructions, and imaged at DIV16–28 either live or after fixation.

#### Non-neuronal Cell Cultures and Transfections

COS-7 cells were obtained from ATCC and grown in DMEM supplemented with 10% FBS, 100 U/mL penicillin, and 100mg/mL streptomycin at 37°C in humidified atmosphere at 5% CO_2_. They were seeded on glass bottom matTek dishes at least 16 hours prior to transfection, which was performed by Lipofectamine 2000 per manufacturers specifications. All cultured cells were routinely tested for mycoplasma contamination and found to be negative.

## METHOD DETAILS

### Generation of Plasmids

Most constructs were generated with regular PCR and/or restriction enzyme digestion and ligation. Some constructs were ligated using In-Fusion Cloning (Takara Bio). Details of primer sets, plasmids, and cDNA clones used for each construct can be found in Key resources table.

All constructs were sequenced in their entirety before use in any experiment.

### Live Cell Imaging and Immunofluorescence

Confocal and AiryScan imaging were performed using LSM880, LSM800 (Carl Zeiss Microscopy) microscopes with 63X/1.40 NA plan-apochromat oil immersion objective and 32-channel gallium arsenide phosphide (GaAsP)-photomultiplier tubes (PMT) area detector. For CLEM, light microscopy was performed with an Andor DragonFly microscope with either a PlanApo 63X/1.4 NA oil immersion objective or a 20X air objective and equipped with a Zyla cMOS camera. 405nm, 488 nm, 561 nm and 633 laser lines were used.

#### Live cell imaging.

Non-neuronal cells were imaged using either culturing media or Live Cell Imaging buffer, and cultured neurons were imaged in modified Tyrode Buffer (119mM NaCl, 5mM KCl, 2mM CaCl_2_, 2mM MgCl_2_, 30mM glucose, 10mM HEPES, pH = 7.35).

#### Immunofluorescence.

Neurons were fixed with 4% PFA, 4% sucrose, 1mM MgCl_2_ and 0.1mM CaCl_2_ in PBS. COS-7 cells were fixed in 4% PFA in PBS. In both cases fixation was carried out for 20 min at room temperature. Cells were then washed 3x with PBS and incubated sequentially in i) permeabilization buffer (0.1% saponin in PBS) for 10 mins, ii) blocking buffer (2% BSA in PBS) for 1 hour at room temperature or overnight at 4 °C and iii) with primary antibodies in blocking buffer overnight at 4 °C or phalloidin in blocking buffer for 1hr at room temperature. Samples were washed 3x in PBS and incubated with Alexa Fluor-conjugated secondary antibodies (1:500 in the blocking buffer) for an hour at room temperature followed by 3x wash in PBS.

### Correlative Light and Electron Microscopy (CLEM)

Cells were plated on 35mm grid, glass-bottom MatTek dishes P35G-1.5–14-CGRD), infected or transfected as indicated, fixed in 2.5% glutaraldehyde in 0.1M cacodylate buffer, and imaged by light microscopy. They were subsequently postfixed in 2% OsO_4_ and 2% K_4_Fe(CN)_6_ (Sigma-Aldrich, St. Louis, MO) in 0.1M sodium cacodylate buffer, *en bloc* stained with 2% aqueous uranyl acetate, dehydrated and embedded in Embed 812. Regions of interest were sectioned (50–60nm) and imaged using a Talos L120C TEM microscope at 80kV. EM reagents are from EMS, Hatfield, PA unless noted otherwise.

## QUANTIFICATION AND STATISTICAL ANALYSIS

### Image Analysis

Image analysis was performed using FIJI. For measuring the distances between sheets, publicly available InteregeDistance macro by Santosh Patnaik was used. Plots were prepared using MATLAB. For presentation purposes brightness and contrast of images were adjusted, and Noise > Despeckle function of FIJI was applied to some images. Line scan plots were generated by drawing a line across the image with a width of 10 points and using Plot Profile function of FIJI. To determine the correlation between synaptopodin-ER protein and the size of stacks, the maximum intensity of this fluorescent construct was measured, and the maximum number of sheets in the corresponding stack was also determined.

## Supplementary Material

1

2 **Video S1. Retention of synaptopodin association with ER upon hypotonic stress. Related to** Figure 2. COS-7 cell expressing mRFP-synaptopodin (magenta) and the ER marker, EGFP-MOSPD1, is exposed to a hypotonic solution. Note that the majority of the synaptopodin inclusions remain associated with the vesiculated ER. Time after exposure to the hypotonic solution is shown.

3 **Video S2. FIB-SEM stack of COS-7 cell expressing synaptopodin-ER. Related to** Figure 5. The stacks of cisternae induced by the expression of this fusion construct are highlighted in magenta. The large dark spheres are lipid droplets.

## Figures and Tables

**KEY RESOURCES TABLE:** 

REAGENT or RESOURCE	SOURCE	IDENTIFIER
Antibodies		
Rabbit polyclonal anti-Synaptopodin (SE-19)	Sigma-Aldrich	Cat# S9442, RRID:AB_261570
Mouse monoclonal Ankyrin-G (Clone 106/65)	NeuroMab	Cat# 75–147, RRID:AB_10675130
MAP2	Thermo Fisher Scientific	Cat# PA1–10005, RRID:AB_1076848
Critical commercial assays		
Takara Bio In-Fusion seamless cloning	Takara Bio	Cat# 638949
Bacterial and virus strains		
pAAV-HA-EGFP-Synpo	Penn Vector Core	N/A
Chemicals, peptides, and recombinant proteins		
Alexa Fluor^™^ 488 Phalloidin	Thermo Fisher Scientific	Cat# A12379
Latrunculin A	Sigma-Aldrich	Cat# 428026
Hybernate-A Medium	Thermo Fisher Scientific	Cat# A1247501
HBSS, no Calcium, no Magnesium	Thermo Fisher Scientific	Cat# 14170112
DNase I, Grade II, from bovine pancreas	Sigma-Aldrich	Cat# 10104159001
Papain	Worthington Biochemical Corporation	Cat# LS003127
B27 Supplement (50X), serum free	Thermo Fisher Scientific	Cat# 17504044
Glutamax^™^ Supplement	Thermo Fisher Scientific	Cat# 35050061
DMEM, high glucose	Thermo Fisher Scientific	Cat# 11965126
Fetal Bovine Serum, dialyzed, US origin	Thermo Fisher Scientific	Cat# 26400044
Penicillin-Streptomycin (10,000 U/mL)	Sigma-Aldrich	Cat# 15140122
Lipofectamine 2000 Transfection Reagent	Life Technologies	Cat# 11668019
Live Cell imaging solutions	Thermo Fisher Scientific	Cat# A14291DJ
HEPES	American Bio	Cat# ab00892–00100
16% Paraformaldehyde Aqueous Solution, EM Grade	Electron Microscopy Sciences	Cat# 15710
Glutaraldehyde Aqueous 25%, EM Grade	Electron Microscopy Sciences	Cat# 16220
Neurobasal^™^-A Medium	Thermo Fisher Scientific	Cat# 10888022
Clontech Labs 3P CALPHOS MAMMALIAN TRANSF KIT	Takara	Cat# 631312
L-Cysteine hydrochloride	Sigma-Aldrich	Cat# C7477
Poly-D-lysine hydrobromide	Sigma-Aldrich	Cat# P0899
Cytochalasin D	Sigma-Aldrich	Cat# C2618
Experimental models: Cell lines		
COS-7 cell	IZSLER	Cat# BS CL 103, RRID:CVCL_0224
Experimental models: Organisms/strains		
C57BL/6J	The Jackson Laboratory	RRID:MGI:3028467
129-Synpo^tm1Mndl/J^	The Jackson Laboratory	RRID:IMSR_JAX:028822
Oligonucleotides		
Primers for cloning mRFP-FKBP12-Synaptopodin: Primer 1: ACCGGCGCCttgtacaccggactcagatctcgaagc Primer 2: agtccggacttgtacaattccagttttagaagctccacatc	This paper	N/A
Primers for cloning mRFP- SynaptopodinΔ(384–473): Primer 1: tgaccccggagctctatgcccgccgc Primer 2: agagctccggggtcaccttgggcttctcc	This paper	N/A
Primers for cloning mRFP-FKBP12-Synaptopodin (1–383): Primer 1: TGACCCCGTAACACACCGCGGGCCCG Primer 2: TGTGTTACGGGGTCACCTTGGGCTTCTCC	This paper	N/A
Primers for cloning mRFP-FKBP12-Synaptopodin (474–690): Primer 1: TCGACTTCGAGCTCTATGCCCGCCGC Primer 2: AGAGCTCGAAGTCGACTGCAGAATTCGAAGC	This paper	N/A
Primers for cloning mRFP-FKBP12-Synaptopodin ΔCalsarcin: Primer 1: CCAATCAGTCCTGGAAGTACACCACTAACGC Primer 2: TCCAGGACTGATTGGGTTTGGGCTTCGG	This paper	N/A
Primers for cloning mRFP-FKBP12-Synaptopodin (380–690): Primer 1: tcgacttcGTGACCCCGAATCCAGATTTGC Primer 2: GGGTCACGAAGTCGACTGCAGAATTCGAAGC	This paper	N/A
Primers for cloning mRFP-FKBP12-Synaptopodin(380–690) ΔCalsarcin: Primer 1: CCAATCAGTCCTGGAAGTACACCACTAACGC Primer 2: TCCAGGACTGATTGGGTTTGGGCTTCGG	This paper	N/A
Primers for cloning mRFP-CG1674: For CG1674: Primer 1: GCAGTCGACTTCATGGATTCTACTTTAAATATTGAGA ATG Primer 2: GCCCGCGGTGTGTTAAAAATCAGAGTACGGTAGATTTC For Backbone (mRFP-Synaptopodin): Primer 1: TAACACACCGCGGGCCCG Primer 2: CATGAAGTCGACTG CAGAATTCGA	This paper	N/A
Primers for cloning mRFP-Synaptopodin2L: For Synaptopodin2L: Primer 1: GCAGTCGACTTCATGGGTGCTGAGGAGGAGGTGC Primer 2: GCCCGCGGTGTGTTACAACTGGTGCCCTGCCCC For Backbone (mRFP-Synaptopodin): Primer 1: TAACACACCGCGGGCCCG Primer 2: CATGAAGTCGACTGCAGAATTCGA	This paper	N/A
Primers for cloning α-actinin-2-AcGFP-Sec61β: Primer 1: CGCTAGCGCTACCGGATGAACCAGATAGAGCCCGGC Primer 2: CATGGTGGCGACCGGTAGATCGCTCTCCCCGTAGAG	This paper	N/A
Recombinant DNA		
Plasmid: HA-EGFP-Synaptopodin	Falahati et al. ^14^	N/A
Plasmid: EGFP-Magi1	Falahati et al. ^14^	N/A
Plasmid: EGFP-Magi2	Falahati et al. ^14^	N/A
Plasmid: EGFP-Pdlim7	Falahati et al. ^14^	N/A
Plasmid: Synaptopodin-AcGFP-Sec61β	Falahati et al. ^14^	N/A
Plasmid: pAAV-GFP-MCS	De Camilli Lab	N/A
Plasmid: Kinectin1-EGFP	De Camilli Lab	N/A
Plasmid: GFP-CAAX	De Camilli Lab	N/A
Plasmid: EGFP-Atlastin1	De Camilli Lab	N/A
Plasmid: Lunapark-mCherry	De Camilli Lab	N/A
Plasmid: EGFP-MOSPD1	Tomasetto Lab	Addgene plasmid #226404
Plasmid: STIM1-RFP	De Camilli Lab	N/A
Plasmid: VAP-B-mCherry	De Camilli Lab	N/A
Plasmids: pAAV-HA-EGFP-Synaptopodin, mRFP-FKBP12-Synaptopodin, mRFP-SynaptopodinΔ(384–473), mRFP-FKBP12-Synaptopodin (1–383), mRFP-FKBP12-Synaptopodin (474–690), mRFP-FKBP12- Synaptopodin ΔCalsarcin, mRFP-CG1674, mRFP-Synaptopodin2L, α-actinin-2-AcGFP-Sec61β, HA-SynpoLong-mCherry-Sec61β, HA-Synpo2L-mCherry-Sec61β, HA-CG1674-mCherry-Sec61β	This paper	N/A
cDNA CG1674	Drosophila Genomics Resource Center	IP15312
mRFP-synaptopodin	A. Triller [Institut de Biologie de I’Ecole Normale Supérieure (IBENS), Paris, France]	N/A
EGFP-α-actinin-2	Hall et al. ^63^	Addgene plasmid #52669
EGFP-MyosinIIA	Jacobelli et al. ^64^	Addgene plasmid #38297
pAcGFP-Sec61β	Zuleger et al. ^51^	Addgene plasmid #62008
mRFP-FKBP	Varnai et al. ^65^	Addgene plasmid #67514
pCMV-DeAct-SpvB	Harterink et al. ^60^	Addgene plasmid #89446
Venus/Cerulean-Synaptopodin-A in pcDNA6	Morris et al. ^66^	Addgene plasmid #190798
Reticulon 4-GFP and GFP-Climp63	Schroeder et al. ^56^	N/A
cDNA Synpo2L	DNASU	DNASU: HsCD00861847
Software and algorithms		
ImageJ	Schneider et al.^7^	https://imagej.nih.gov/ij/
MATLAB	MathWorks	RRID:SCR_001622
FIJI	NIH	RRID:SCR_002285

## Highlights

- Synaptopodin (Synpo) can crosslink the endoplasmic reticulum to actin bundles
- ER–targeted Synpo makes spine apparatus-like structures (SALs) in COS-7 cells
- SAL formation requires F-actin and is independent of other ER-shaping proteins
- SALs are molecularly distinct ER subdomains
